# Supplementary material for: A Bayesian semiparametric approach for inference on the population partly conditional mean from longitudinal data with dropout
Source: Biostatistics. 2021 Apr 21;24(2):372–87. doi: 10.1093/biostatistics/kxab012 (PMC10102880; doi:10.1093/biostatistics/kxab012)
Supplement: kxab012_Supplementary_Data [file kxab012_supplementary_data.pdf]

Supplementary material - A Bayesian semi-parametric approach for inference on the population partly conditional mean from longitudinal data with dropout

JOSEFSSON, MARIA[1,2,3,*], DANIELS, MICHAEL J.[4], & PUDAS, SARA[3,5]

[1]Department of Statistics, USBE, Umeå University, Sweden.

[2]Centre for Demographic & Ageing Research, Umeå University, Sweden.

[3]Umeå Center for Functional Brain Imaging, Umeå University, Sweden.

[4]Department of Statistics, University of Florida, USA.

[5]Department of Integrative Medical Biology, Umeå University, Sweden.

[*]Corresponding author, E-mail: maria.josefsson@umu.se

APPENDIX A, OTHER STRATEGIES FOR ESTIMATING THE PPCM

In the simulation study and the empirical data example we compare our proposed approach to four other estimators for estimating the PPCM: MB-lm, MRP, HT and GREG.

The first estimator (MB-lm) is a parametric version of our semi-parametric approach, where the working models are specified as Bayesian additive linear regression models instead of using DART.

The second approach is multilevel regression and poststratification (MRP). The MRP specifies a multilevel model for the outcome at time t, using random (or modeled) effects for some of the predictors. That is, the working model is replaced by the following multilevel regression model

$$y_{it} = \alpha + \sum_{m=0}^{t-1} \beta_m y_{im} + \sum_{m=0}^{t} \sum_{k=1}^{K} b_{x_{mk}} + \varepsilon_{it},$$

where α is the fixed intercept, $\beta_0, \ldots, \beta_{t-1}$ are the fixed effects for the outcome history, and $b_{x_1 j_{x_k}}, \ldots, b_{x_K j_{x_k}}$ are the random effects for the j_{x_k}th category of the auxiliary variable

x_k, where $k = 1, \dots, K$, and $j_{x_k} = 1, \dots, J_{x_k}$. All random effects are modeled using independent normal distributions, such that $b_{x_k} \sim N(0, \sigma^2_{x_k})$, $k = 1, \dots, K$, and, $\varepsilon_{it} \sim N(0, \sigma^2_{MRP_t})$. In the second step, the multilevel model is used for making predictions $\hat{y}_{it}$ from $\bar{y}_{it-1}$ and $\bar{\mathbf{x}}_{it}$, for all $i \in U$ given $\bar{s}_{it} = 1$, and is (similarly to our approach) obtained by integrating over the outcome history $\bar{y}_{t-1}$. The poststratification estimate for the population mean is given by $\text{PPCM}_t^{MRP} = \frac{1}{\sum_{i \in U} s_{it}} \sum_{i \in U} \hat{y}_{it} s_{it}$.

The third approach is an extension of the classic Horvitz-Thompson weighting estimator (HT), $\hat{\mu}^{HT} = \frac{1}{N} \sum_{i \in c} \frac{y_i}{\pi_i}$. In a longitudinal setting with MAR missingness and death, the HT estimator is obtained by replacing π_i with the probability of participation at time t given survival at that time point, here denoted by w_{it}. The PPCM_t is given by,

$$\text{PPCM}_t^{HT} = \frac{1}{\sum_{i \in U} s_{it}} \sum_{i \in c} \frac{y_{it}}{w_{it}} \sum_{i \in c} r_{it} s_{it},$$

where

$$w_{it} = \pi_i \prod_{k=0}^{t} \Pr(r_{ik} = 1 \mid \bar{y}_{ik-1}, \bar{r}_{ik-1}, \bar{x}_{ik}, \bar{s}_{ik} = 1)$$

for all $i \in c$. The response mechanism, the second term on the right hand side, can be estimated from data using e.g. a logistic regression model. In the empirical study using the Betula data, two separate datasets are used for finite population inference and the participation probability is not known. Then π_i must be estimated from data using cell weight adjustment. Cell weight adjustment classifies the sample and population into distinct post-stratification cells based on the auxiliary variables recorded for both groups. The sampled participants in cell j are weighted by the inverse of the sampled rate in cell j. That is, for individual $i \in j$, $\hat{\pi}_i = n_j/N_j$, where n_j is the number of individuals in cell j in the sample and N_j is the number of individuals in cell j in the population.

However, difficulties may arise in this setting. For example, continuous variables have to be dichotomized, and further, with a large set of auxiliary variables, the cell sample sizes can be small. This may result in biased and unstable estimates. To overcome the latter, weight trimming and cell collapsing is recommended.

The fourth approach is a dual-modelling strategy that combines prediction and weighting. The general regression estimator (GREG) at time t is given by,

$$\text{PPCM}_t^{GREG} = \frac{1}{\sum_{i \in U} s_{it}} \left[\sum_{i \in U} \hat{y}_{it} + \sum_{i \in c} r_{it} s_{it} \left(\frac{y_{it} - \hat{y}_{it}}{\hat{w}_{it}} \right) \sum_{i \in c} r_{it} s_{it} \right].$$

The approach require both a model for the outcome and the participation mechanism, and is double robust in the sense that it remains consistent if either one of the models are correctly specified.

Appendix B, ICD codes

The following ICD codes extracted from the in-patient and cause of death registers were considered: Dementia, ICD8, ICD-9: 290 and ICD-10: F00 - F01, F03. CVD, ICD8, ICD-9: 410, 411, 412, 413, 414, 428, 430, 431, 432, 433, 434, 435, 436, 437, 438, 440, 441, 442, 443, 444, 445, 446, 447, 448, and ICD-10: I21, I60 - I63. Diabetes, ICD8, ICD-9: 250, and ICD-10: E10 - E14. Depression, ICD8, ICD-9: 296, and ICD-10: F32 - F33. Alcohol substance abuse, ICD8, ICD-9: 291, and ICD-10: F10.
